# A genomics approach to understanding the role of auxin in apple (*Malus *x *domestica) *fruit size control

**DOI:** 10.1186/1471-2229-12-7

**Published:** 2012-01-13

**Authors:** Fanny Devoghalaere, Thomas Doucen, Baptiste Guitton, Jeannette Keeling, Wendy Payne, Toby John Ling, John James Ross, Ian Charles Hallett, Kularajathevan Gunaseelan, GA Dayatilake, Robert Diak, Ken C Breen, D Stuart Tustin, Evelyne Costes, David Chagné, Robert James Schaffer, Karine Myriam David

**Affiliations:** 1 The New Zealand Institute for Plant & Food Research Limited (PFR), Private Bag 92169, Auckland 1142, New Zealand; 2 School of Biological Sciences, University of Auckland, Private Bag 92019, Auckland 1142, New Zealand; 3 PFR, Private Bag 11600, Palmerston North 4442, New Zealand; 4 School of Plant Science, University of Tasmania, GPO Box 252-55, Hobart, Tasmania 7001, Australia; 5 PFR, Private Bag 1401, Havelock North 4157, New Zealand; 6 PFR, Old Mill Road, RD3, Motueka 7198, New Zealand; 7 INRA, UMR AGAP, Equipe Architecture et Fonctionnement des Espèces Fruitières, Avenue Agropolis - TA-A-108/03, 34398 Montpellier Cedex 01, France

## Abstract

**Background:**

Auxin is an important phytohormone for fleshy fruit development, having been shown to be involved in the initial signal for fertilisation, fruit size through the control of cell division and cell expansion, and ripening related events. There is considerable knowledge of auxin-related genes, mostly from work in model species. With the apple genome now available, it is possible to carry out genomics studies on auxin-related genes to identify genes that may play roles in specific stages of apple fruit development.

**Results:**

High amounts of auxin in the seed compared with the fruit cortex were observed in 'Royal Gala' apples, with amounts increasing through fruit development. Injection of exogenous auxin into developing apples at the start of cell expansion caused an increase in cell size. An expression analysis screen of auxin-related genes involved in auxin reception, homeostasis, and transcriptional regulation showed complex patterns of expression in each class of gene. Two mapping populations were phenotyped for fruit size over multiple seasons, and multiple quantitative trait loci (QTLs) were observed. One QTL mapped to a region containing an Auxin Response Factor (*ARF106*). This gene is expressed during cell division and cell expansion stages, consistent with a potential role in the control of fruit size.

**Conclusions:**

The application of exogenous auxin to apples increased cell expansion, suggesting that endogenous auxin concentrations are at least one of the limiting factors controlling fruit size. The expression analysis of *ARF106 *linked to a strong QTL for fruit weight suggests that the auxin signal regulating fruit size could partially be modulated through the function of this gene. One class of gene (*GH3*) removes free auxin by conjugation to amino acids. The lower expression of these *GH3 *genes during rapid fruit expansion is consistent with the apple maximising auxin concentrations at this point.

## Background

The hormonal control of fruit growth and development has been well established across many different plants. One hormone, auxin, has been shown to control the initial growth and expansion of tissues following fertilisation [1,2] and inhibit ripening. Early work with strawberry and other fruits proposed a mechanism whereby auxin produced by the developing seed regulated fruit growth by controlling firstly cell division and secondly cell expansion. As the seeds subsequently mature, auxin concentrations drop, acting as a signal for ripening to proceed. Supporting this mechanism is the observation that applied auxins can induce parthenocarpy in fruits such as tomato [3], fruit size in peach [4], cell enlargement in cherry [5] and delay ripening in strawberry [1]. Developmental regulation by the principal auxin in higher plants, IAA (Indole Acetic Acid), is achieved through the coordination of complex processes: auxin metabolism (involving biosynthesis, conjugation and catabolism), auxin transport (long distance and polarised auxin transport) and auxin signalling (perception, transduction and response). The balance of synthesis, breakdown, conjugation and transport is tightly regulated, leading to auxin homeostasis [6].

*De novo *auxin synthesis in plants results from multiple pathways dependent or independent of tryptophan [7,8]. IAA can be conjugated to amino acids, sugars and methyl esters. Enzymes that conjugate IAA to amino acids are encoded by members of the group II of the *GH3 *(*Gretchen Hagen 3*) family of auxin-induced genes [9]. Very little is known about the role of *GH3 *genes during fruit development. However, it has recently been shown in grape that *GH3.1 *plays a role in the formation of IAA-Asp late during development, coinciding with the onset of ripening [10]. Release of IAA from IAA conjugates is achieved by hydrolytic cleavage [11]. Auxin transport from sites of synthesis to target cells is complex and highly regulated, playing a crucial role in both establishing and changing homeostasis. Auxin is transported both passively through the vasculature and actively through transporters [12]. The most characterised auxin transport family is the efflux carrier PIN family. PIN proteins are vital for normal plant development. Mutations in the *PIN1 *gene lead to pin-like organs with no development of flower parts in *Arabidopsis thaliana *(*Arabidopsis*) [13] and members of the *PIN *family are highly expressed early during tomato fruit development, suggesting a role during fruit set [14].

The current model for auxin perception and signalling involves two types of receptors [15,16]: the Auxin Binding Protein 1 (ABP1), located at the plasma membrane, and the Transport Inhibitor 1/Auxin signalling F-Box family (TIR1/AFB), a set of nuclear receptors [17-19]. ABP1 is involved in very early auxin responses leading, for example, to the modification of ion fluxes [20]. ABP1 has been shown to be essential for plant life (a mutation in *ABP1 *in *Arabidopsis *is lethal) and is important for both cell division and cell expansion [21-23]. However, the details of the pathway going through ABP1 are poorly understood. In tomato, the *diageotropica *(*dgt*) mutant displays many auxin-related developmental defects and fruit development is dramatically altered, with a reduced fruit size [24]. *DGT *encodes a cyclophilin, known to act as signalling intermediate, and was shown to use ABP1 as an extracellular receptor for auxin-dependent cell growth response [25]. The signalling pathway involving TIR1 is now well characterised and explains most of auxin-regulated gene expression [16]. The three families of early auxin responsive genes, *Aux/IAA, GH3 *and *SAUR (Small Auxin Up Regulated)*, contain a binding motif to the ARF transcription factor (Auxin Response Factor). At low auxin concentrations, a heterodimer of an ARF and an Aux/IAA protein represses transcription. At higher auxin concentration, auxin will bind to TIR1/AFB, an F-box protein that is part of an SCF complex (Skp1/Cullin/F-box), and triggers the degradation of the repressor Aux/IAA through the 26S proteasome. This will ultimately release the ARF transcription factor to modulate expression of early auxin response genes. In fleshy fruits, most of our knowledge involving the ARF-Aux/IAA complex during fruit development comes from studies in tomato. *SlARF7 *is expressed in placental and ovule tissues and down-regulated soon after pollination. Silencing of the *SlARF7 *gene leads to parthenocarpic fruit development, showing that *SlARF7 *functions as a negative regulator of fruit set [26]. Similarly, silencing of the *SlIAA9 *gene expression also confers parthenocarpy [27]. *SlARF4 *(also known as DR12) seems to play a role later in fruit development, as its expression increases throughout tomato fruit development, with the highest levels in early red-stage fruit. Down-regulation of *SlARF4 *leads to pleiotropic phenotypes including dark-green immature fruit, enhanced firmness and unusual cell division in the fruit pericarp, which is thicker than in wild-type (WT) fruits [28,29].

While many fleshy fruits are carpel derived, the fruit from *Malus *x *domestica *(apple) is unusual, as it is derived from the hypanthium, a tube of fused sepals, petals and anther derived tissue. However, like other fruits, apple development can be separated into periods of cell division, cell expansion, maturity, and ripening [30]. While there have been a few studies on auxin content in apple [31,32], there is little research reported on the role of auxin in apple fruit development at the molecular level. There are a large number of different cultivars of apples showing a range of different flowering times, maturity times and times to ripen. One cultivar, 'Royal Gala', is a naturally occurring sport of the 'Gala' cultivar. It is a mid-season apple, and its growth and development has been well characterised. 'Royal Gala' has been the subject of a number of genomics studies, including a large-scale expressed sequencing tag (EST) sequencing project [33] and a microarray study of the fruit development [30] and fruit ripening [34]. It is readily transformable, with transgenic apples for *ACO1 *suppression [34,35], *MYB10 *overexpression [36], and *POLYGALACTURONASE 1 *[37] being reported. Recently a parent of 'Royal Gala', 'Golden Delicious', has had its genome sequenced [38].

Here we have investigated the role of auxin on apple fruit development and assessed the expression of genes involved in homeostasis, transport and signalling of auxin. The location of auxin-related genes in the genome sequence of apple was compared with QTLs for fruit weight, which is linked to fruit size.

## Results

### Changes in auxin content over fruit development

Apple fruit have previously been given four distinct developmental stages following pollination, consisting of Stage 1 (cell division), Stage 2 (cell expansion), Stage 3 (fruit maturity) and Stage 4 (ripening) [30]. In 'Royal Gala' apples, Stage 1 takes 0-25 days after full bloom (DAFB), rapid growth (Stage 2) covers 20-60 DAFB, after which the fruit continue to grow at a slower rate as the fruit matures (Stage 3), with the ripening process starting around 132 DAFB (Stage 4), with tree-ripe eating apples at 146 DAFB [30].

**Figure 1:**
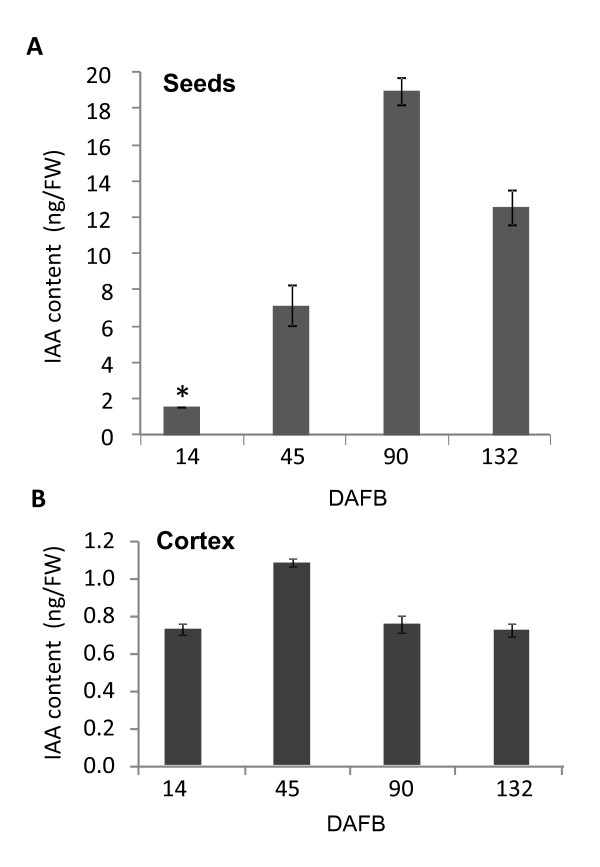
**Free auxin content in apple tissue**. Changes in free IAA (Indole Acetic Acid) content (ng/g fresh weight; FW) in the seed (A) and cortex (B) of developing 'Royal Gala' apples over fruit development (DAFB: Days After Full Bloom). Measurements were performed on 4 and 2 biological replicates for cortex and seed samples respectively. * indicates seed sample for which only one extraction was possible. Error bars represent standard error of the mean.

To investigate the role of auxin during fruit development, the free IAA amounts were measured at representative times (14, 45, 90 and 132 DAFB) during the different fruit development stages in 'Royal Gala' fruit cortex and seed. IAA concentrations in the seed showed a large increase during fruit development, reaching a maximum concentration of 19 ng/g fresh weight (FW) (Figure 1A). The IAA concentrations in the fruit cortex were considerably lower than in the seed, ranging from 0.6 to 1 ng/g FW, with a significant increase during cell expansion (Figure 1B). This is consistent with the literature, showing high auxin concentrations in the seeds of tomato and strawberry, compared with those in the fruits [2,39].

### Effect of auxin treatment on apple fruit growth

**Figure 2:**
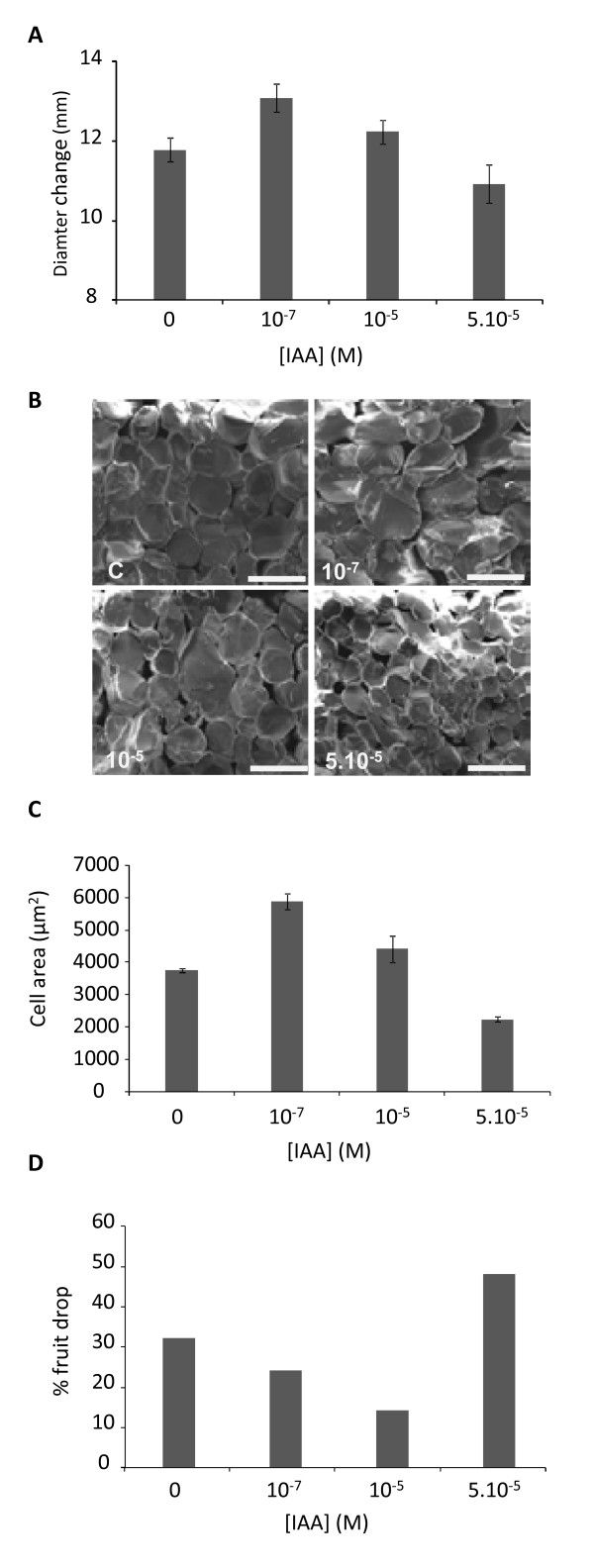
**The effect of auxin on cell expansion**. Indole Acetic Acid (IAA) was injected at different concentrations (10^-7 ^M, 10^-5 ^M and 5.10^-5 ^M) through the calyx of 'Royal Gala' apples 30 days after full bloom. Two weeks following injection, diameter increase was measured (A), Cryo-Scanning Electron Microscopy photographs of five representative fruits were taken (scale bar: 100 μm) (B), average cell area was calculated (C) and the percentage of abscised fruit was determined (D). Fifty fruit were injected per concentration. Error bars represent standard error of the mean.

As there was an increase in auxin in the cortex tissue during Stage 2 (the rapid cell expansion phase), we assessed the effect of injecting three auxin concentrations within the physiologically active range for auxins (10^-6 ^M) into fruit at the beginning of Stage 2 (30 DAFB). The growth of each apple was assessed by recording the diameter of the fruit at injection and after two weeks of subsequent growth. During the two-week period, all fruit showed an increase in fruit size (Figure 2A). The two lowest auxin concentrations (10^-7 ^M and 10^-5 ^M) caused a significant increase in fruit diameter compared with the control (Figure 2A), while the highest concentration (5.10^-5 ^M) appeared to inhibit fruit growth. The increased fruit growth observed with the 10^-7 ^M and 10^-5 ^M treatments corresponded to a greater increase in the cell size (Figure 2B,C) compared with that of control apples. During the early stages of apple development, there is a natural self-thinning event. Apples typically have 5 florets per cluster, which for commercial purposes are thinned to 1-2 fruit per cluster, depending on the localised fruit load. Two to three apples were chosen per cluster for injection and the rest were hand thinned. During the two-week treatment, the control showed a 32% fruit drop. When injected with auxin at 10^-7 ^M and 10^-5 ^M, a higher degree of fruit retention was observed, with only 14% fruit dropped in the 10^-5 ^M treatment. Additional auxin promoted fruit drop, with 48% fruit abscised from the 5.10^-5 ^M concentration (Figure 2D).

### Genomic screening of auxin-related genes in the apple genome

The apple draft genome [38] was screened for six classes of auxin-related genes. These included the receptor-like genes *ABP1 *and *TIR1*/*AFB*, the transcriptional control genes *ARF *and *Aux*/*IAA*, and the auxin homeostasis genes, the *PIN *genes and *GH3*-like genes. All six classes of genes searched were well represented in the apple genome (Table 1, full list in Additional file 1). The apple genome has been shown to have undergone a genome duplication, so the numbers of genes were compared with the numbers of auxin-related genes in the recently published woodland strawberry genome [40] (a related diploid Rosaceae species) (Summarised in Table 1, and a full list in Additional file 2). In each gene family, there are approximately twice the numbers of auxin-related genes in apple than in strawberry.

**Table 1:** Numbers of auxin-related genes in apple compared with strawberry and *Arabidopsis*

Class	Apple	Strawberry	*Arabidopsis*
*ABP1*	2	1	1

*TIR1/AFB*	8	4	5

*ARF*	37	18	23

*Aux/IAA*	40	26	29

*PIN*	11	9	8

*GH3 *(Group II)	15	9	10

**Figure 3:**
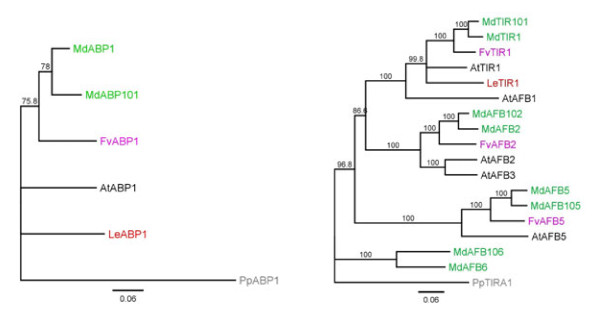
**Phylogenetic tree of the auxin receptors**. (A) ABP1 class of receptors, (B) TIR1/AFB class of receptors. Maximum alignable protein sequences of the auxin receptors from apple (green), strawberry (lilac), *Arabidopsis *(black) and tomato (red) were aligned using MUSCLE and phylogenetic trees were built using neighbour joining. Bootstraps of 1000 iterations are given. *At: Arabidopsis thaliana, Fv: Fragaria vesca, Md: Malus *x *domestica, Pp: Physcomitrella patens, Sl: Solanum lycopersicum*

**Figure 4:**
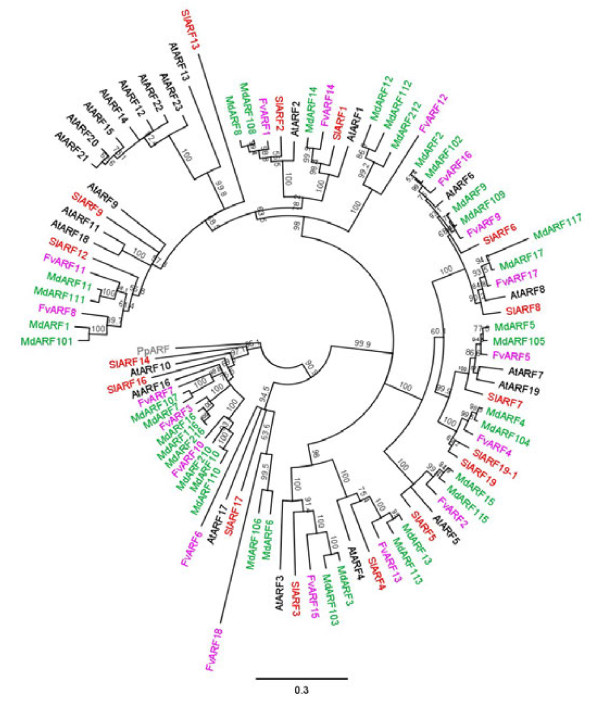
**Phylogenetic tree of the auxin response factors**. The DNA binding domains of the auxin response factors (ARF) from apple (green), strawberry (lilac), *Arabidopsis *(black) and tomato (red) were aligned using MUSCLE and phylogenetic trees were built using neighbour joining. Bootstraps of 1000 iterations are given. *At: Arabidopsis thaliana, Fv: Fragaria vesca, Md: Malus *x *domestica, Pp: Physcomitrella patens, Sl: Solanum lycopersicum*

A phylogenetic analysis of the six classes of genes using the predicted protein sequence from apple, strawberry, tomato and *Arabidopsis *was performed for each class of genes (Figures 3, 4 Additional files 2, 3). In each of the phylogenetic clusters, the majority of the apple genes were contained in subclades consisting of a single strawberry gene. These subclades typically had two apple duplicated genes for each strawberry gene, with the occasional subclade showing a single apple gene, or three apple genes per strawberry gene. This 2:1 ratio of genes was robustly observed in the *ABP1, TIR1/AFB *and *ARF *class of genes, with Aux/*IAA, GH3 *and *PIN *showing a less robust pattern with some strawberry and apple genes showing no corresponding related sequences. The predicted location of the two apple duplicates genes were often found on homeologous chromosomes identified in the apple genome [38] (chromosomal locations are given in Additional file 1), with duplicated genes found on non-homeologous chromosomes occurring 18% of the time. Because of this tight phylogenetic relationship between the strawberry genes and two apple genes, when possible both apple homologues were named after the already annotated strawberry genes, for example *ARF1 *of strawberry clustered with genes in apple that were labelled *ARF1 *and *ARF101*. This nomenclature was not followed if the gene had previously been published or released in GenBank. With these genes, the existing name was kept (Additional file 1).

While the gene families from the two Rosaceae species, strawberry and apple, were tightly aligned with each other, there was considerable divergence from the *Arabidopsis *genes. For example, there is evidence of clade expansion in the *ARF *genes in *Arabidopsis *including the *AtARFs 12, 14, 15, 20, 21, 22, 23 *(Figure 4). The *TIR1*-like proteins also suggests small family expansion in *Arabidopsis *(Figure 3). Small subclades containing only proteins from strawberry, apple and tomato were also observed.

### Expression analysis of auxin-related genes during apple fruit development

**Figure 5:**
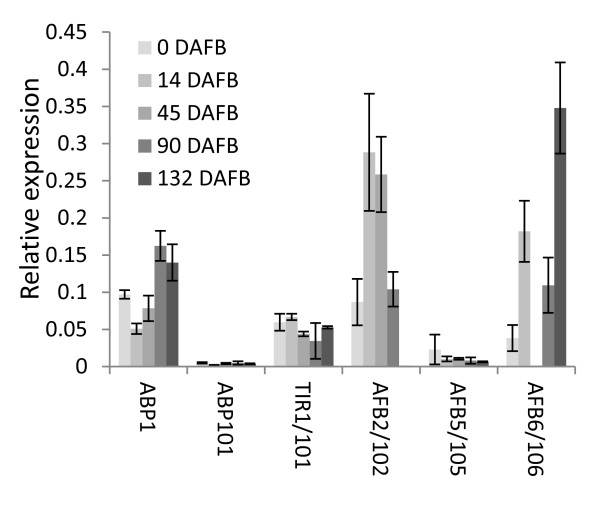
**Expression analysis of auxin receptor class of genes**. Expression analysis (by qPCR) for the auxin receptors *ABP1 *and *TIR1/AFB *class of genes are shown through five stages of fruit development (0: fruit set, 1: cell division, 2: cell expansion, 3: fruit maturation and 4: fruit ripening) represented by fruit harvested at 0, 14, 45, 90, 132 Days After Full Bloom (DAFB). Where the primers were unable to distinguish between the homeologous genes, both gene names are given. Expression is relative to actin, with error bars representing standard error of the mean (n = 4)

**Figure 6:**
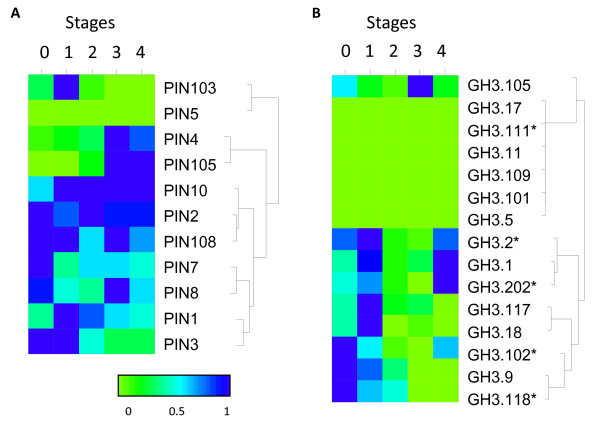
**Expression analysis of auxin homeostasis genes**. Expression analysis (by qPCR) of auxin-conjugating enzyme (*GH3*) and transport genes (*PIN*) over fruit development are grouped by hierarchical clustering, normalised to maximum expression of each gene. Five stages of fruit development (0: fruit set, 1: cell division, 2: cell expansion, 3: fruit maturation and 4: fruit ripening) represented by fruit harvested at 0, 14, 45, 90, 132 Days After Full Bloom (DAFB). Asterisks represent primer pairs unable to distinguish between homeologous genes.

**Figure 7:**
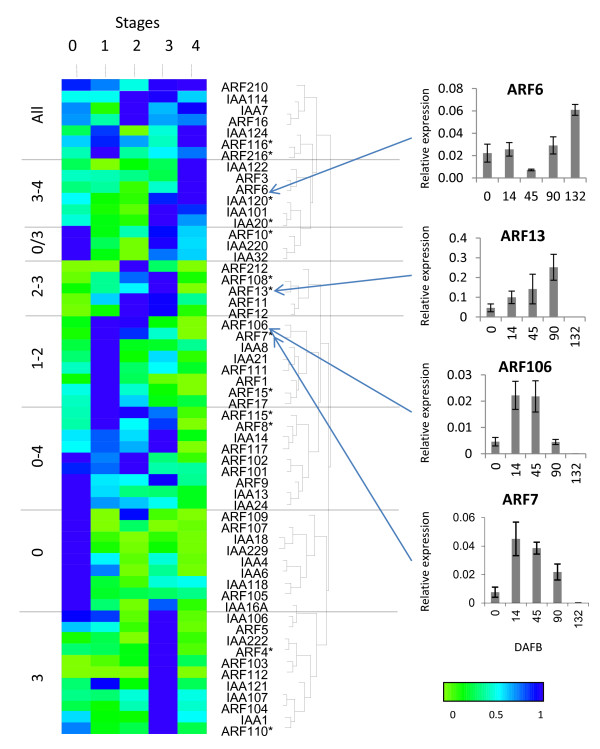
**Expression analysis of auxin transcriptional regulators**. Expression of the transcriptional regulators *ARF *and *Aux/IAA *class of genes are clustered according to expression patterns over fruit development, grouped by hierarchical clustering, normalised to maximum expression of each gene. Five stages of fruit development (0: fruit set, 1: cell division, 2: cell expansion, 3: fruit maturation and 4: fruit ripening) are represented by fruit harvested at 0, 14, 45, 90, 132 Days After Full Bloom (DAFB). Asterisks represent primer pairs unable to distinguish between homeologous genes. The expression patterns of selected genes within the cluster are also presented. Expression is relative to actin, with error bars representing 4 replicates.

Gene expression for each of the auxin-related genes was screened using quantitative polymerase chain reaction (PCR) across flowering and at time points representing the four stages of apple fruit development (0, 14, 45, 90, 132 DAFB) (Additional file 4). Some of the homeologous genes showed very little sequence divergence at the DNA level, making it hard to select optimal qPCR primers that were specific for each gene in the homeologous pairs. Of the 108 genes tested, 25 primer pairs were predicted to be unable to differentiate between the homeologues (Additional file 4). In these instances, the expression patterns are given with both names (Figure 5) or marked with an asterisk (Figures 6, 7).

The genes were divided into three main functional groups for analysis: receptors (Figure 5), homeostasis (Figure 6) and response (Figure 7). When it was possible to differentiate the homeologous genes, the expression patterns within each pair were compared with each other. There were a considerable number of homeologous genes with different expression patterns and also instances where one of the homeologous genes was apparently switched off, for example *ABP1 *and *ABP101 *(Figure 5). This may be because of the quality of the primer, or actual lack of expression. To address this concern, we examined the EST libraries [33] for ESTs corresponding to the 10 receptor-like genes, and used diagnostic DNA polymorphisms to identify each of the homeologues. From these sequence data, it appears that *ABP1 *is transcribed and *ABP101 *is not (confirming the qPCR data). For the *TIR1*/*AFB *class there was a similar pattern, with *TIR1, AFB102*, and *AFB106 *having ESTs, while *TIR101, AFB2*, and *AFB6 *were not represented, suggesting that the transcription of these genes is suppressed. For the apparent lowly expressed *AFB5 *and *AFB105 *there were no ESTs with which to compare. Both the *ABP1 *and *TIR1 *class of receptor genes were expressed constitutively through fruit development. In the *TIR1*/*AFB *family, *AFB102 *showed a Stage 1 and 2 predominant expression, while *AFB106 *showed a high degree of expression at Stage 4 fruit development (Figure 5).

*PIN103 *showed the highest expression only during the cell division (Stage 1), while *PIN4 *and *PIN105 *showed predominant expression as the fruit progressed into maturity and ripening (Stages 3-4) (Figure 6A). Half the *GH3 *conjugating proteins showed high expression levels early in fruit development - cell division (Stages 0 and 1), while all were suppressed during Stages 2-3 (cell expansion), with a number increasing again as the fruit ripened (Figure 6B).

Many of the *ARF*/*Aux/IAA *family of genes had discrete expression patterns for a single stage of fruit development (Figure 7). Half the differentially expressed genes were predominantly expressed during the early stages of fruit development, with a high proportion of Aux/*IAA *genes being highly expressed at full bloom. As auxin has been shown to be central to the regulation of fruit size, which is determined during cell division and cell expansion, we were particularly interested in profiles that had high expression in Stages 1 and 2. In this cluster, four *ARF *and two *Aux/IAA *had highest expression during Stage 1. While *ARF212 *had peak expression at Stage 2, it had a very low expression level. Only two genes had high expression during both these two stages (*ARF7 *and *ARF106*) (Figure 7), suggesting these two genes may play a role in the control cell division and/or expansion affecting final fruit size.

### QTL mapping for fruit size

To assess regions controlling fruit size in apples, two mapping populations were assessed for fruit weight, as a surrogate for fruit size, over a number of successive years. Fruit weight was phenotyped for a total of 572 and 123 genotypes from 'Royal Gala' × 'Braeburn' (RGxBB) and 'Starkrimson' × 'Granny Smith' (STKxGS) mapping populations. Measurements were made over 2 and 5 years of production, and two and one sites, respectively. Analysis of variance showed that the genotypic (G) and the year (Y) effects for fruit weight were highly significant for each population (*P *values < 2.2e-16). For the RGxBB population, the effects of the growing environment (E; *P *value < 2.2e-16) and its interaction with the genotype (GxE; *P *value < 2.2e-16) were highly significant. Best Linear Unbiased Predictors (BLUPs) independent of year and environment were extracted for each genotype for both studied populations and were used as phenotypic data for QTL detection. Six QTL regions were identified for fruit weight using the RGxBB and STKxGS genetic maps on Linkage Group (LG) 5, 8, 11, 15, 16 and 17 (Table 2, Additional file 5). Two QTL regions were conserved across both segregating populations on LG 8 and LG 15. The explained genetic variability (*R^2^*) for each of the QTLs ranged between 3.9% for a 'Royal Gala' QTL on LG 15 to 17.3% for a 'Granny Smith' QTL on LG 8. The global *R^2 ^*were higher in the STKxGS segregating population (53.9%) than in the RGxBB (18.2%). The QTLs detected in the RGxBB segregating population were not involved in any epistasic effect, whereas the three fruit weight QTLs detected in the STKxGS population on LG 8, 15 and 17 were involved in an epistatic effect.

**Table 2:** Characteristics of the Quantitative Trait Loci (QTLs) detected for fruit weight

QTL name	Apple linkage group	Marker used as co-factor for MQM analysis	LOD	Phenotypic variation explained by single QTL	Interaction with other QTL (epistasis)	Global phenotypic variation explained
**'Royal Gala'**						

Fruit weight_2009	8	GD_SNP00293	3.37	3.0%	-	

Fruit weight_2010	15	GD_SNP01850	4.77	3.7%	n.s.	7.1%
		
	12	GD_SNP00714	4.65	3.6%	n.s.	

Fruit weight_Hawke's Bay	6	GD_SNP00166	3.30	2.8%	GD_SNP00004	12.3%
		
	11	GD_SNP00004	6.35	5.2%	GD_SNP00166	
		
	15	GD_SNP01347	4.12	3.1%	n.s.	

Fruit weight	5	GD_SNP00231	6.03	4.4%	n.s.	18.2%
		
	8	GD_SNP01169	7.21	6.2%	n.s.	
		
	11	GD_SNP00004	7.64	6.1%	n.s.	
		
	15	*MdARF106*	5.43	3.9%	n.s.	

**'Braeburn'**						

Fruit weight_2010	15	GD_SNP01813	4.87	5.9%	-	

Fruit weight_Hawke's Bay	16	GD_SNP02087	6.88	6.5%	-	

Fruit weight	15	GD_SNP01534	9.40	6.9%	n.s.	14.3%
		
	16	LG16_3662305	10.98	8.4%	n.s.	

**'Starkrimson'**						

Fruit weight_2008	8	*CH02g09_SG*	3.52	24.0%	n.s.	24%
		
	15	*MdARF106_SG*	3.99	20.9%	n.s.	

Fruit weight	8	*CH02g09_SG*	7.91	27.3%	n.s.	
		
	15	*MdARF106_SG*	5.45	26.3%	n.s.	

**'Granny Smith'**						

Fruit weight	8	CH05a02y_G	5.75	16.9%	MdARF106_SG* MdLD_G	53.9%
		
	15	*MdCLV1c_SG*	5.23	23.3%	CH05a02y_G* MdLD_G	
		
	17	*MdLD_G*	5.5	15.8%	MdARF106_SG* CH05a02y_G	
						

Characteristics of the QTLs detected on separated parental genetic maps, 'Royal Gala', 'Braeburn' (RGxBB), 'Starkrimson' and 'Granny Smith' (STKxGS) map by Multiple QTL Mapping **(**MQM) mapping for fruit weight. QTL detection was carried out using Best Linear Unbiased Predictor (BLUP) as phenotypic data. Different BLUP values were extracted to represent genetic potential for each genotype (Fruit Weight), fruit weight in a given year calculated from the Genotype × Year (GxY) interaction, and fruit weight in a given environment calculated from the Genotype × Environment (GxE) interaction. For the RGxBB population, BLUP values were extracted for the genotype (Fruit Weight), the interaction G × Y (Fruit Weight_2009 and Fruit Weight_2010), and the interaction G × E (Fruit Weight_Hawke's Bay and Fruit Weight_Nelson). For the STKxGS population, BLUP values were extracted for the genotype (Fruit Weight) and for each year (Fruit Weight_2006, Fruit Weight_2007, Fruit Weight_2008, Fruit Weight_2009 and Fruit Weight_2010). For each QTL, the table displays the marker used as a co-factor for MQM analysis, the LOD score, and the percentage of genetic variation explained by each single QTL (*R^2 ^*). When several QTLs were detected for the same trait, the global *R^2 ^*(the proportion of variation explained by the QTLs) and the interactions between QTLs are indicated.

**Figure 8:**
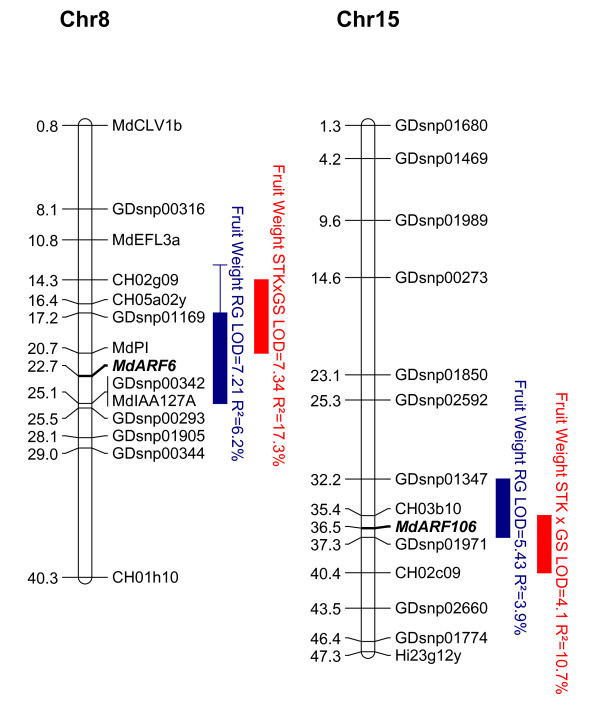
**Quantitative Trait Loci (QTL) for apple fruit size**. Physical positions (Mb) of the Quantitative Trait Loci (QTLs) detected on the parental 'Royal Gala' genetic map (RG, blue) and on the consensus 'Starkrimson' × 'Granny Smith' (STK × GS, red). QTLs are represented by boxes, in which length represents the LOD-1 confidence interval and extended lines represent the LOD-2 confidence interval. Mapped candidate genes *ARF6 *and *ARF106 *are in bold underlined.

The six QTL regions were compared with the *in silico *locations of auxin genes that showed predominant expression during Stages 1 and 2. *ARF106 *is located on LG 15 and could account for the fruit weight QTL on this linkage group. The genetic marker developed from the sequence of *ARF106 *had the highest LOD score (logarithm of odds) for the fruit weight QTL, in both the 'Royal Gala' and 'Starkrimson' × 'Granny Smith' genetic maps (Table 2 and Figure 8). The QTL interval spanned an overlapping area between both STKxGS and RGxBB QTLs, of 1.92 Mb, from markers CH03b10 (35.397 Mb) to GDsnp01971 (37.346 Mb). Within this region, in addition to *ARF106 *(MDP0000232116), 132 other predicted gene models were found (Additional file 6). While some of these genes showed homology to *Arabidopsis *genes that have annotated gene ontology, which may control fruit size, such as involvement in cell division, cell cycle and signal transduction (Additional file 6), these were not studied further as they were outside the scope of this project.

The LG 8 and 15 QTLs were mapped in duplicated regions of the apple genome because of whole genome duplication [38]. The homeologous gene to *ARF106 *is *ARF6*. While expressed later in fruit development, there is still the possibility that *ARF6 *retains some residual fruit size control. The genetic marker developed from *ARF6 *mapped to the fruit size QTL but was not the strongest marker in this region (Table 2 and Figure 8).

## Discussion

With the availability of the whole genome sequence for apple [38], there is now the possibility of identifying the complete gene family for different classes of genes. This powerful resource has been used to identify individual auxin gene classes (mostly *ARF *and *Aux/IAA*) in a number of plants including *Arabidopsis *[41], poplar [42], maize [43,44] and tomato [45]. The auxin signal has been implicated in many components of fruit growth and development, including determination of fruit size through cell expansion, as well as the control of fruit ripening, and the regulation of fruit drop [46]. Here we have presented a genomics screen in apple of different auxin-related genes, covering perception, homeostasis and transcriptional regulation, and their relative expression patterns over fruit development. These expression data provide the ground work for further studies in the role of auxin on apple fruit growth and development.

### Fruit size determination

There are a number of factors that regulate fruit size in apples, those that are controlled by the genetic make-up of the apple (size potential), and those related to the environment in which the apple develops. The environmental control of size is determined by both the effects of orchard husbandry and irrigation and local effects in the apple tree, such as fertilisation success, and localised crop load. We demonstrated that in the RGxBB segregating population, the effect of the year, the environment and its interaction with the genotype were highly significant. Furthermore, a significant difference was observed in the global phenotypic variation explained by the QTLs for fruit size detected in the two segregating populations. The larger explained phenotypic variation for the STKxGS population can be explained by the fact this population was grafted on less dwarfing rootstock ('Pajam') than the RGxBB ('M.9'). In addition, the crop load for the RGxBB was regulated, while the STKxGS was not. While the environment can cause significant amounts of variation, the genetic size potential of the fruit is a major determinant of fruit size. This work and others have shown that size potential is a complex multi-loci trait in apples [47] and other fruits such as tomato [48,49]. The control of fruit size in apples has been linked to both the number of cell division steps that occur directly following pollination and to subsequent cell expansion [50]. Apples have been shown to have a range of different cell sizes across different cultivars [51], and increasing cell number and size through endo-reduplication causes a 38% increase in fruit weight [52]. Our work suggests that auxin signals through this cell division/cell expansion phase may be modulated by an ARF gene (*ARF106*) that is up-regulated during these developmental time points and co-locates with a stable QTL for fruit weight. *ARF106 *is most closely linked to the *AtARF17*, which is microRNA controlled and when over-expressed using a microRNA-resistant form, gives a pleotropic phenotype [53] including excess tissue growth in leaves. The corresponding ARF in tomato *SlARF17 *is expressed highly at fruit ripening (named *SlARF13 *in [45], see Additional file 2).

The balance of auxin is critical for fruit expansion. This auxin signalling is complex, as not only can the presence of auxin elicit a developmental response, but different concentrations can cause different responses [54]. Here we found that injecting different concentrations of auxin could cause an increased cell expansion, decreased fruit growth, and ultimately fruit drop [55]. The increased cell growth with lower amounts of injected auxin suggests that fruit growth is at least in part limited by auxin concentration, as application of more can enhance it, which is consistent with the observations of Percy and collaborators [56].

Extensive molecular research on auxin in *Arabidopsis *has now identified a number of key genes involved in the regulation of auxin content of plant tissue, and the method by which the auxin signal is converted into a developmental change. Part of the complexity of the auxin signal transduction can be explained by the regulation of response genes: by developmentally regulating both the signal transduction transcription factors (ARF) and the modulators of transcription (Aux/IAA), a complex network of regulation can be achieved. This complexity can explain how the same signalling molecule can relay different signals at different times during development [6].

### Auxin-related processes in fruit set

While the molecular control of auxin is best understood in the model plant *Arabidopsis*, which bears dry dehiscent fruit, there has been a substantial body of work in fleshy fruit species. Surprisingly, for many of the ARF proteins, the tomato orthologue (Asterid) is more closely related to the apple gene than the *Arabidopsis *orthologue (a Rosid, like apple). The ARF proteins, which are more similar to those in tomato (MdARF1, 101, 11, 111, 8, 108, 4, 104, 13 113, 3, 103) are all expressed at a specific stage in fruit development (Figure 7). This may suggest a strong evolutionary pressure for conservation within fleshy fruit species. This conservation is also observed in the expression patterns presented in [45], with genes such as *MdARF113/SlARF4, MdARF4/SlARF19 *expressed at similar times in fruit development. The phylogenetic discrepancy is also observed in the IAA cluster (Additional File 3); however, there are currently no published genomics studies of IAA genes in tomato to make this comparison.

Key auxin-related genes such as *AtARF8 *(FWF) in *Arabidopsis *and *SlARF7 *in tomato have been associated with fruit set [26,57]. Mutations in *AtARF8 *and down-regulation of *SlARF7 *cause parthenocarpic fruit in *Arabidopsis *and tomato respectively, indicating that *AtARF8 *is functionally equivalent to *SlARF7*, while it is not the closest homologue (Figure 4) [58]. Down-regulation of *SlIAA9 *also leads to development of parthenocarpy and it has been hypothesised that it could work in the same pathway as *SlARF7 *[59]. *SlARF7 *clusters with *ARF5 *and *ARF105, AtARF8 *clusters with *ARF17 *and *ARF117*, while *SlIAA9 *clusters with *IAA8, IAA27A *and *IAA127A *(Figure 4, Additional file 3). Of these apple genes, *ARF105 *has highest expression at full bloom, like *SlARF7 *[26,45] (named *SlARF9 *in this later study), suggesting that this may play a similar role, while *ARF5 *is undetectable.

### Auxin and ripening

The role of auxin in the regulation of ripening in strawberry has been well established after it was found that application of auxins through the peduncle caused a significant delay in ripening [1]. This link suggesting that auxin is a negative regulator of ripening was further enforced by a study over-expressing a pepper *GH3*-related gene in tomato [60]. In this study, the *CcGH3 *over-expressing lines matured earlier than untransformed lines and ripened earlier with the addition of exogenous ethylene. The closest homologue from of this *CcGH3 *in apple is *GH3.1*, which also shows a large increase in expression around fruit maturity (Figure 6B). Interestingly, this gene is also expressed during the cell division stage. As well as the *GH3*-related genes, there is also a cluster of auxin signal transduction genes that are up-regulated at Stage 4 (Figure 7), suggesting that an auxin signal can still be transduced at this stage. This is consistent with auxin playing a role in fruit maturation and ripening that is well known in non-climacteric fruits [1,10] and beginning to be established in climacteric fruit [61].

Another gene in tomato *SlIAA3 *is induced by both ethylene and auxin at fruit maturation [62]. *SlIAA3 *clusters with *IAA6 *and *IAA106*, neither of which is expressed at the ripening stage. However, 6 *ARF/Aux/IAA *genes are highly expressed at ripening, suggesting a similar role in apple. Down-regulation of another tomato auxin response factor, *SlARF4 *(also referred initially as DR12) [28,29], leads to unripe fruit that are firmer because of a perturbed pectin metabolism, and the fruit display an unusual cell division pattern in the pericarp. *SlARF4 *clusters with *ARF13 *and *ARF113*, of which *ARF13 *is expressed during cell expansion and maturation. This differs slightly from *SlARF4*, which shows increasing expression during tomato fruit development, with the highest in ethylene-producing fruits.

## Conclusions

This work has provided a genomics study of auxin regulation in apples, with many auxin-related genes changing through fruit development. The complexity of expression patterns of these genes suggests a complex role of auxin regulation in apple fruit development. Exogenously applied auxin during the end of cell division/early cell expansion phase can increase fruit size, showing that auxin is at least in part one of the limiting factors controlling cell expansion. The role of one auxin response gene, *ARF106*, which maps to a size-related QTL, needs to be further investigated to determine if this gene plays a role in apple fruit size regulation.

## Methods

### Selection of genes in the apple genome

Auxin-related genes were selected by using BLASTP search of known *Arabidopsis *auxin-related genes against predicted apple protein sequences within the 'Golden Delicious' whole genome sequence [38]. The predicted MDP numbers were mapped to chromosome location using the apple *Malus *x *domestica *GBrowse from the Genome Database for Rosaceae (http://www.rosaceae.org/gb/gbrowse/malus_x_domestica/) (Additional file 1). The protein sequences were compared with strawberry and *Arabidopsis *genes based on phylogeny. For each gene family, the longest highly conserved alignable protein sequence regions were used as detailed below: ABP1: the whole protein without the leader peptide. TIR1/AFB: The F-box domain and the leucine rich repeat domains. ARFs: The DNA binding domain. AUX/IAA domains I-IV. PINs: Transmembrane domains. GH3: The whole protein. Apple auxin-related genes were named firstly on published GenBank accessions and secondly on the nearest strawberry-named gene in the strawberry predicted protein sequences V2. Alignments and phylogenetic trees were generated using the Geneious Pro™ 5.4 (Biomatters). Multiple alignments were performed using MUSCLE and phylogenetic trees were built using neighbour joining with 1000 bootstraps. Sequences from the moss *Physcomitrella patens *were used to root the trees. Accession numbers of proteins from *Arabidopsis*, strawberry and tomato are given in Additional file 2.

### Auxin injection in apples and assessment

Developing apple fruit were injected with different concentrations of IAA (Sigma-Aldrich, UK), dissolved in a 0.1% ethanol solution, 30 days after full bloom. Control apples were injected with a 0.1% ethanol solution. For each treatment, 100 μL were injected through the calyx using a syringe, into a minimum of 50 apple fruit; the apple diameter was measured on its equator, and the fruit tagged. Fifteen days after injection, the apples were harvested and the apple diameter was re-measured to establish the growth rate. For each treatment, the cortex tissue adjacent to the calyx from five representative apples was assessed using freeze fracture scanning electron microscopy (SEM). CryoSEM was performed using a Polaron PP2000 Cryo Transfer system (Quorum Technologies, Ringmer UK) attached to an FEI Quanta250 Scanning Electron Microscope (FEI Hillsboro OR). Blocks of apple tissue about 4 × 6 × 2 mm were placed in aluminium sample holders, held in brass transfer shuttles, using a mixture of colloidal graphite and OCT™ compound (Sakursa Finetek, Zoeterwoude, NL) as adhesive, so that a portion of the apple protruded from the surface. These were frozen in liquid nitrogen slush. Samples were transferred under vacuum to the PP2000 preparation stage, which was held at -150°C and the apple tissue fractured using a cooled metal blade or probe. The fractured surface was sputter-coated with gold/palladium (60 sec) and transferred to the SEM for observation on a stage cooled to -150°C using an accelerating voltage of 15 kV. Cell size was measured by counting the number of whole cells in the fracture window from each of the five treatment samples.

### Auxin content measurement

IAA was extracted from 'Royal Gala' cortex (4 replicates) and seed (2 replicates) at different times during fruit development. Tissue samples were homogenised and IAA was extracted with 80% (v/v) methanol containing 250 mg l^-1 ^butylated hydroxytoluene. 10 ng of [^13 ^C_6_]IAA, internal standard was added to extracts and left at 4°C for 24 h. The extracts were then filtered through Whatman no. 1 filter paper. Samples were reduced in volume to less than 1 mL under vacuum at 35°C and an aliquot was loaded onto a Sep-Pak C18 cartridge in 0.4% acetic acid. IAA was eluted with 50% methanol in 0.4% acetic acid. The eluate was dried and taken up in 1% acetic acid. Samples were then analysed using a Waters Acquity H-series UPLC coupled to a Waters Xevo triple quadrupole mass spectrometer. A Waters Acquity UPLC BEH C18 column (2.1 × 100 mm × 1.7 μm particles) was utilised. The solvents were 1% acetic acid in water (Solvent A) and acetonitrile (Solvent B) at a flow rate of 0.25 mL/min, with a linear gradient from 80% A:20% B to 50% A:50% B at 4.5 mins, followed by re-equilibration to starting conditions for 3 mins. Five μL of each sample was injected. The mass spectrometer was operated in positive ion electrospray mode with a needle voltage of 2.4 KV, and selected reaction monitoring was used to detect IAA and^13 ^C_6 _IAA. The ion source temperature was 150°C, the desolvation gas was nitrogen at 1000 L/h, the cone gas flow was 50 L/h and the desolvation temperature was 300°C. The MS/MS transitions monitored were m/z 176.2 to 130.1 for IAA and 182.2 to 136.1 for^13 ^C_6 _IAA. Cone voltage was 18 V and collision energy was 18 V. Dwell time was 161 ms per channel.

Data were analysed using MassLynx software. IAA and^13 ^C_6 _IAA eluted at 3.74 mins under these conditions.

### Gene expression analysis

'Royal Gala' apple fruit were harvested in the year 2006-07 from the Plant & Food Research Orchard, Hawke's Bay. For flowers, whole flowers were sampled; all fertilised apples had seed removed before harvesting. As the apples developed, sections of tissue from at least 10 apples were harvested (containing skin, cortex and core) into liquid nitrogen. RNA extraction and cDNA was generated as described in [30]. Gene expression was measured using quantitative PCR (qPCR) using Power SYBR^® ^green probes (Applied Biosystems, UK). Primers were used that gave a single melting peak for each of the genes assessed (Primer sequences can be found in Additional file 4). Quantitative PCR was conducted across two instruments: *ARF *and *TIR *gene expression were performed on a Roche LightCycler^® ^480™ with the set up according to [63], whereas *ABP1, PIN, GH3 *and *Aux/IAA *were assessed using an ABI PRISM^® ^7900 HT Sequence Detection System (Applied Biosystems) according to the method described in [64]. In all cases, *actin *was used as a reference gene.

### Mapping fruit size

Two F_1 _progenies were used to study the fruit size. The first contained a duplicated population of 590 seedlings from a 'Royal Gala' and 'Braeburn' cross (RGxBB), grafted onto 'M.9' rootstock, located at two locations in New Zealand (PRF research orchards in Havelock North and Motueka). The second contained a duplicated population of 123 seedlings from a 'Starkrimson' and 'Granny Smith' cross (STK×GS) grafted on the semi-dwarfing rootstock 'Pajam 1' located in France (Melgueil INRA Montpellier Experimental station). For the RGxBB seedlings, two seasons of apples were assessed. Apples were thinned to a low crop load of 4 fruit per cm^2 ^of trunk size and a minimum of five representative fruit sizes were weighed at harvest. For the STK×GS population, no thinning was undertaken. Five seasons of total apple crop on each tree were harvested weighed and counted, and the average weight of apple calculated per year.

Analysis of variance and linear models (in R software v.2.9.2 - R Development Core Team, 2009 [65]) were used to assess genetic and environmental regulation of fruit size at each site over the number of seasons measured. QTL analyses were performed using the RGxBB parental genetic maps and on the STKxGS consensus map [66,67]. JoinMap 3.0 [68] was used for constructing linkage maps. Fruit weight QTL intervals for each population were defined based on the peaks LOD-1 and LOD-2.

For additional gene mapping, PCR primer pairs were designed using Primer 3 Plus software (http://www.bioinformatics.nl/cgi-bin/primer3plus/primer3plus.cgi), to select 100-200 bp fragments spanning a putative SNP (Single Nucleotide Polymorphism). High Resolution Melting (HRM) analysis was used for the detection of DNA polymorphisms [69], performed on a LightCycler 480^®^, as described in [70].

The list of predicted gene transcripts present within the QTL interval was extracted from GDR (http://www.rosaceae.org/projects/apple_genome) and we present the results of pairwise comparison of the *Malus *x *domestica *genome predicted genes against the *Arabidopsis *TAIR10_pep_20100802 database using BLASTP with an EXP cut-off < 1e-30.

## Authors' contributions

Experimental procedures were performed by FD, TD, WP, JK, KG (extracted RNA, screened the genome for auxin-related genes and performed expression analysis). JRR, TJL measured auxin content, ICH undertook the microscopy assessment, KCB, GAD harvested apples and assessed the fruit, GAD, RD, DST set up the RGxBB cross and conducted fruit weight assessments on this population BG, EC, and DC constructed the genetic map and identified QTLs for fruit weight. RJS and KMD conceived the project and analysed the data; DC, RJS and KMD wrote the paper. All authors read and approved the final manuscript.

## Supplementary Material

Additional file 1 **List of auxin-related genes in apples**. Table of Predicted Apple genes by MDP number [38], designated names and chromosome location (Gene and protein sequences can be obtained from GDR: http://www.rosaceae.org). Click here for file

Additional file 2 **Accession numbers of proteins sequences from other species used to build phylogenetic tree**. Click here for file

Additional file 3 **Phylogenetic trees for PIN, GH3 and Aux/IAA class of genes**. Protein sequences of PIN, GH3, Aux/IAA from apple (green), strawberry (lilac), *Arabidopsis *(black) and tomato (red) were aligned using MUSCLE and phylogenetic trees were built using neighbour joining. Bootstraps of 1000 iterations are given. *At: Arabidopsis thaliana, Fv: Fragaria vesca, Md: Malus *x *domestica, Pp: Physcomitrella patens, Sl: Solanum lycopersicu*. Click here for file

Additional file 4 **List of qPCR primers used to measure gene expression patterns, and relative expression used for clustering**. Click here for file

Additional file 5 **Mapping Quantitative Trait Loci (QTL)s for fruit weight in RGxBB and STKxGS**. Click here for file

Additional file 6 **Genes identified within QTL interval for fruit weight on Ch15**. List of the 133 predicted gene transcripts present within the QTL interval for fruit weight on LG15. The table displays gene location and putative function based on homology to known genes in *Arabidopsis thaliana *(pairwise comparison of the *Malus *x *domestica *genome predicted genes against the *Arabidopsis *TAIR10_pep_20100802 database using BLASTP with an EXP cut-off < 1e-30). Data were retrieved from the Genome Database for Rosaceae (http://www.rosaceae.org/projects/apple_genome). Click here for file
