# Supplementary material for: A genomics approach to understanding the role of auxin in apple (Malus x domestica) fruit size control
Source: BMC Plant Biol. 2012 Jan 13;12:7. doi: 10.1186/1471-2229-12-7 (PMC3398290; doi:10.1186/1471-2229-12-7)
Supplement: Additional file 1 — List of auxin-related genes in apples. Table of Predicted Apple genes by MDP number [38], designated names and chromosome location (Gene and protein sequences can be obtained from GDR: http://www.rosaceae.org). [file 1471-2229-12-7-S1.DOC]

**LIST OF AUXIN RELATED GENES FOUND IN APPLE (*Malus x domestica*)**

Gene accessions were retrieved from the Genome Database for Rosaceae (GDR: <http://www.rosaceae.org/>).

Duplicated genes that are not on homeologous linkage group are in red.

**Receptors**

| **Gene name** | **Lg** | **Apple ID (GDR)** | **Genbank** | **Position (Mb)** |
| --- | --- | --- | --- | --- |
| MdABP1 | 3 | MDP0000707508 | AAB47752 | 31.668 |
| MdABP101 | 11 | MDP0000459936 | - | 33.599 |
|  |  |  |  |  |
| MdTIR1 | 3 | MDP0000125975 | - | 31.992 |
| MdTIR101 | 11 | MDP0000498419 | - | 33.959 |
| MdAFB2 | 9 | MDP0000268652 | - | 0.444 |
| MdAFB102 | 17 | MDP0000203334 | - | 0.312 |
| MdAFB5 | 15 | MDP0000809218 | - | 37.587 |
| MdAFB105 | 0 | MDP0000135966 | - | - |
| MdAFB6 | 10 | MDP0000305861 | - | 29.215 |
| MdAFB106 | 5 | MDP0000255696 | - | 3.479 |

**Homeostasis**

| **Gene name** | **Lg** | **Apple ID** | **Genbank** | **Position (Mb)** |
| --- | --- | --- | --- | --- |
| MdPIN1 | 14 | MDP0000138035 | EF406255/EF406256 | 28.405 |
| MdPIN10 | 6 | MDP0000200231 | EF406260/EF406261 | 23.865 |
| MdPIN2 | 0 | MDP0000119864 | - | - |
| MdPIN3 | 1 | MDP0000156440 | - | 2.567 |
| MdPIN103 | 12 | MDP0000784665 | - | 8.283 |
| MdPIN4 | 16 | MDP0000234528 | EF406257 | 0.183 |
| MdPIN7 | 13 | MDP0000497581 | EF406258/EF406259 | 1.552 |
| MdPIN5 | 4 | MDP0000297331 | - | 19.946 |
| MdPIN105 | 12 | MDP0000322329 | - | 31.293 |
| MdPIN8 | 9 | MDP0000250518 | - | 2.797 |
| MdPIN108 | 17 | MDP0000301544 | - | 3.153 |
|  |  |  |  |  |
| MdGH3.1 | 5 | MDP0000873893 | - | 26.384 |
| MdGH3.101 | 9 | MDP0000209432 | - | 14.779 |
| MdGH3.2 | 1 | MDP0000612660 | - | 12.471 |
| MdGH3.102 | 15 | MDP0000872868 | - | 25.163 |
| MdGH3.202 | 15 | MDP0000666539 | - | 25.175 |
| MdGH3.5 | 3 | MDP0000132162 | - | 25.579 |
| MdGH3.105 | 11 | MDP0000402444 | - | 25.963 |
| MdGH3.9 | 11 | MDP0000204381 | - | 34.287 |
| MdGH3.109 | 3 | MDP0000568498 | - | 32.492 |
| MdGH3.11 | 1 | MDP0000193700 | - | 10.712 |
| MdGH3.111 | 7 | MDP0000786650 | - | 16.301 |
| MdGH3.17 | 4 | MDP0000834656 | - | 7.078 |
| MdGH3.117 | 13 | MDP0000226842 | - | 10.072 |
| MdGH3.18 | 5 | MDP0000811081 | - | 12.159 |
| MdGH3.118 | 17 | MDP0000214081 | - | 6.398 |

**Transcriptional regulation**

**ARF family**

| **Gene name** | **Lg** | **Apple ID** | **Genbank** | **Position (Mb)** |
| --- | --- | --- | --- | --- |
| MdARF1 | 7 | MDP0000194603 | ADL36575 | 17.537 |
| MdARF101 | 1 | Not annotated | - | - |
| MdARF2 | 5 | MDP0000232417 | ADL36576 | 7.677 |
| MdARF102 | 5 | MDP0000268306 | - | 7.058 |
| MdARF3 | 10 | MDP0000179650 | ADL36577 | 27.962 |
| MdARF103 | 5 | MDP0000173151 | - | 4.743 |
| MdARF4 | 16 | MDP0000134824 | - | 19.215 |
| MdARF104 | 13 | MDP0000185253 | - | 31.009 |
| MdARF5 | 2 | MDP0000886637 | - | 7.275 |
| MdARF105 | 15 | MDP0000876321 | - | 15.832 |
| MdARF6 | 8 | MDP0000256621 | - | 22.707 |
| MdARF106 | 15 | MDP0000232116 | - | 36.502 |
| MdARF7 | 3 | MDP0000221322 | - | 10.462 |
| MdARF107 | 11 | MDP0000274442 | - | 11.536 |
| MdARF8 | 6 | MDP0000310875 | - | 16.525 |
| MdARF108 | 14 | MDP0000258032 | - | 20.297 |
| MdARF9 | 17 | MDP0000153538 | - | 11.147 |
| MdARF109 | 9 | MDP0000319906 | - | 10.973 |
| MdARF10 | 8 | MDP0000190950 | - | 6.258 |
| MdARF110 | 8 | MDP0000156207 | - | 6.271 |
| MdARF210 | 1 | MDP0000319072 | - | 13.999 |
| MdARF11 | 2 | MDP0000139073 | - | 18.996 |
| MdARF111 | 15 | MDP0000259062 | - | 13.265 |
| MdARF12 | 11 | MDP0000138860 | - | 33.860 |
| MdARF112 | 3 | MDP0000123466 | - | 31.900 |
| MdARF212 | 3 | MDP0000138853 | - | 31.920 |
| MdARF13 | 11 | MDP0000412781 | - | 10.937 |
| MdARF113 | 15 | MDP0000225980 | - | 25.040 |
| MdARF14 | 8 | MDP0000929655 | - | 29.301 |
| MdARF15 | 8 | MDP0000211459 | - | 1.177 |
| MdARF115 | 12 | MDP0000143749 | - | 21.353 |
| MdARF16 | 12 | MDP0000167246 | ACI13681 | 15.812 |
| MdARF116 | 4 | MDP0000750392 | - | 8.274 |
| MdARF216 | 4 | MDP0000291384 | - | 8.267 |
| MdARF17 | 17 | MDP0000550049 | - | 23.864 |
| MdARF117 | 9 | MDP0000294251 | - | 30.780 |

**Aux/IAA family**

| **Gene name** | **Lg** | **Apple ID** | **Genbank** | **Position (Mb)** |
| --- | --- | --- | --- | --- |
| MdIAA1 | 12 | MDP0000945260 | ABI36486* | 30.230 |
| MdIAA101 | 4 | MDP0000295589 | - | 21.376 |
| MdIAA4 | 8 | MDP0000123816 | - | 6.624 |
| MdIAA6 | 5 | MDP0000324398 | - | 14.261 |
| MdIAA106 | 10 | MDP0000176753 | - | 19.773 |
| MdIAA7 | 10 | MDP0000010086 | ABI36487* | 19.786 |
| MdIAA107 | 5 | MDP0000237499 | - | 14.276 |
| MdIAA8 | 1 | MDP0000580010 | ABI36492* | 7.408 |
| MdIAA13 | 12 | MDP0000255223 | - | 10.429 |
| MdIAA113 | 9 | MDP0000164095 | - | 18.989 |
| MdIAA14 | 9 | MDP0000211848 | - | 19.755 |
| MdIAA114 | 17 | MDP0000262602 | - | 15.804 |
| MdIAA16A | 12 | MDP0000663301 | ABI36493* | 30.238 |
| MdIAA16B | 4 | MDP0000363509 | ABI36494 | 21.364 |
| MdIAA18 | 13 | MDP0000303142 | - | 21.548 |
| MdIAA118 | 16 | MDP0000208345 | - | 16.289 |
| MdIAA19 | 16 | MDP0000124810 | - | 16.443 |
| MdIAA119 | 13 | MDP0000213864 | - | 21.624 |
| MdIAA20 | 5 | MDP0000132805 | - | 15.666 |
| MdIAA120 | 10 | MDP0000250876 | - | 17.566 |
| MdIAA220 | 10 | MDP0000284467 | - | 17.551 |
| MdIAA21 | 0 | MDP0000270789 | - | - |
| MdIAA121 | 16 | MDP0000131759 | - | 9.903 |
| MdIAA22 | 8 | MDP0000753736 | - | 14.103 |
| MdIAA122 | 15 | MDP0000267601 | - | 3.174 |
| MdIAA222 | 15 | MDP0000130583 | - | 3.174 |
| MdIAA24 | 4 | MDP0000211934 | - | 6.703 |
| MdIAA124 | 15 | MDP0000246042 | - | 44.401 |
| MdIAA25 | 8 | MDP0000296324 | - | 6.520 |
| MdIAA27A | 15 | MDP0000090281 | ABI36495* | 39.374 |
| MdIAA127A | 8 | MDP0000456476 | - | 25.116 |
| MdIAA27B | 2 | MDP0000174664 | ABI36497* | 4.846 |
| MdIAA127B | 0 | MDP0000801571 | - | - |
| MdIAA28 | 4 | MDP0000151877 | - | 23.035 |
| MdIAA29 | 2 | MDP0000157035 | - | 2.743 |
| MdIAA129 | 15 | MDP0000195460 | - | 10.243 |
| MdIAA229 | 8 | MDP0000543718 | - | 18.938 |
| MdIAA32 | 13 | MDP0000277775 | - | 2.080 |
| MdIAA132 | 16 | MDP0000299826 | - | 0.875 |
| MdIAA33 | 13 | MDP0000146848 | - | 33.903 |

* partial genbank sequence
