# Supplementary material for: A genomics approach to understanding the role of auxin in apple (Malus x domestica) fruit size control
Source: BMC Plant Biol. 2012 Jan 13;12:7. doi: 10.1186/1471-2229-12-7 (PMC3398290; doi:10.1186/1471-2229-12-7)
Supplement: Additional file 2 — Accession numbers of proteins sequences from other species used to build phylogenetic tree. [file 1471-2229-12-7-S2.DOCX]

**Protein sequenceS USED to build phylogenetic trees**

*Arabidopsis* sequences correspond to the TAIR accession, strawberry (*Fragaria vesca*) gene numbers have been obtained from the strawberry genome server (<http://www.strawberrygenome.org/>) and tomato (*Solanum lycopersicum*) proteins correspond to Genbank accessions except for Aux/IAA for which sequences have been retrieved from the Sol Genomics Network ([http://solgenomics.net/](http://solgenomics.net/%20) ).

**ABP1 sequences**

*Arabidopsis thaliana:* AT4G02980

*Fragaria vesca:* 1536826

*Solanum lycopsersicum:* Q9ZRX6

**TIR/AFB sequences**

*Arabidopsis thaliana:* AtTIR1: AT3G62980; AtAFB1: AT4G03190; AtAFB2: AT3G26810; AtAFB3: AT1G12820; AtAFB5: AT5G49980

*Fragaria vesca:* FvTIR1: 1534045; FvAFB2: 1510591; FvAFB5: 1521187

*Solanum lycopersicum:* SlTIR1: ACU81102

**PIN sequences**

*Arabidopsis thaliana:* AtPIN1: AT1G73590; AtPIN2: AT5G57090; AtPIN3: AT1G70940; AtPIN4: AT2G01420; AtPIN5: AT5G16530; AtPIN6: AT1G77110; AtPIN7: AT1G23080; AtPIN8: AT5G15100

*Fragaria vesca:* FvPIN1: 1518508; FvPIN2: 1521435; FvPIN3: 1509687; FvPIN4: 1513196; FvPIN5: 1525903; FvPIN8: 1510482; FvPIN6: 1515202; FvPIN9: 1514559; FvPIN10: 1521433

*Solanum lycopersicum:* SlPIN1: ADR30406; SlPIN2: ADR30409; SlPIN3: ADR30411(=BAJ09456: SlPIN3 in reference [12]); SlPIN4: ADR30410 (=BAJ09455: SlPIN2 in reference [12]) ; SlPIN5: ADR30412; SlPIN6: ADR30414; SlPIN7: ADR30408; SlPIN8: ADR30415; SlPIN9: ADR30407(=BAJ09454: SlPIN1 reference [12]); SlPIN10: ADR30413

**GH3 sequences**

*Arabidopsis thaliana*: AtGH3.1: AT2G14960; AtGH3.11: AT2G46370; AtGH3.12: AT5G13320; AtGH3.17: AT1G28130; AtGH3.2: AT4G37390; AtGH3.3: AT2G23170; AtGH3.4: AT1G59500; AtGH3.5: AT4G27260; AtGH3.6: AT5G54510; AtGH3.9: AT2G47750

*Fragaria vesca:* FvGH3.1: 1531943; FvGH3.2: 1533108; FvGH3.5: 1512391; FvGH3.6: 1539797; FvGH3.9: 1519688; FvGH3.11: 1528016; FvGH3.12: 1536837; FvGH3.17: 1532131; FvGH3.18: 1519409

**ARFs sequences**

*Arabidopsis thaliana*

AtARF1: AT1G59750; AtARF2: AT5G62000; AtARF3: AT2G33860 ; AtARF4: AT5G60450; AtARF5: AT1G19850; AtARF6: AT1G30330; AtARF7: AT5G20730 ; AtARF8: AT5G37020; AtARF9: AT4G23980; AtARF10: AT2G28350; AtARF11: AT2G46530; AtARF12: AT1G34310; AtARF13: AT1G34170; AtARF14: AT1G35540; AtARF15: AT1G35520; AtARF16: AT4G30080; AtARF17: AT1G77850; AtARF18: AT3G61830; AtARF19: AT1G19220; AtARF20: AT1G35240; AtARF21: AT1G34410; AtARF22: AT1G34390; AtARF23: AT1G43950

*Fragaria vesca*

FvARF1: 1515559; FvARF2: 1517616; FvARF3: 1518857; FvARF4: 1521345; FvARF5: 1522038; FvARF6: 1525161; FvARF7: 1525954; FvARF8: 1528247; FvARF9: 1531834; FvARF10: 1532383; FvARF11: 1534221; FvARF12: 1536889; FvARF13: 1537642; FvARF14: 1538352; FvARF15: 1538867; FvARF16: 1539489; FvARF17: 1540726; FvARF18: 1532177

*Solanum lycopersisum*

| **Genbank** | | **Other references** | | |
| --- | --- | --- | --- | --- |
| **Name** | **Accession** | **Chaabouni et al., 2009**  **[57]** | **Kumar et al., 2011**  **[41]** | **De Jong et al., 2009**  **[24]** |
| SlARF1 | ADJ10892 | SlARF1 | SlARF1 |  |
| SlARF2 | ABC69711 | SlARF2 | SlARF5 |  |
| SlARF3 | ABC69710 | SlARF3 | SlARF3 |  |
| SlARF4 | ABC69715 | SlARF4 | SlARF14 |  |
| SlARF5 | ADP0665 | SlARF5 |  |  |
| SlARF6 | ADK91822/ACU30063 | SlARF6 | SlARF10 |  |
| SlARF7 | ABO33637 |  | SlARF9 | SlARF7 |
| SlARF8 | ABS83388 | SlARF8 | SlARF4 |  |
| SlARF9 | ADH03013 |  | SlARF12 |  |
| SlARF10 | ADK26472 |  | SlARF15 |  |
| SlARF12 | ADP06660 |  |  |  |
| SlARF13 | ADP06661/ADP06662 |  | SlARF7 |  |
| SlARF14 | ADP06664 |  |  |  |
| SlARF16 | ADJ96591 |  |  |  |
| SlARF17 | ADR66030 |  | SlARF13 |  |
| SlARF19 | ADI87602/ADN28050 | SlARF7 | SlARF8 |  |
| SlARF19-1 | ADP06663 |  |  |  |
|  |  |  | SlARF6 |  |
|  |  |  | SlARF16 |  |
|  |  |  | SlARF17 |  |

**Aux/IAA sequences**

*Arabidopsis thaliana*

AtIAA1 : AT4G14560; AtIAA10 : AT1G04100; AtIAA11 : AT4G28640; AtIAA12 : AT1G04550; AtIAA13 : AT2G33310; AtIAA14 : AT4G14550; AtIAA15 : AT1G80390; AtIAA16 : AT3G04730; AtIAA17 : AT1G04250; AtIAA18 : AT1G51950; AtIAA19 : AT3G15540; AtIAA2 : AT3G23030; AtIAA20 : AT2G46990; AtIAA26 : AT3G16500; AtIAA27 : AT4G29080; AtIAA28 : AT5G25890; AtIAA29 : AT4g32280; AtIAA3 : AT1G04240; AtIAA30 : AT3G62100; ATIAA31 : AT3G17600; AtIAA32 : AT2G0120; AtIAA33 : AT5G57420 ; AtIAA34 : AT1G15050 ; AtIAA4 : AT5G43700 ; AtIAA5 : AT1G15580 ; AtIAA6 : AT1G52830 ; AtIAA7 : AT3G23050; AtIAA8: AT2G22670; AtIAA9: AT5G65670

*Fragaria vesca*

FvIAA1: 1525680 ; FvIAA2 : 1525682 ; FvIAA3 : 1514738 ; FvIAA4 : 1515118 ; FvIAA5 : 1515330 ; FvIAA6 : 1517315 ; FvIAA7 : 1517460 ; FvIAA8 : 1520746 ; FvIAA9 : 1520982 ; FvIAA11 : 1514300 ; FvIAA12 : 1529407 ; FvIAA13 : 1534821 ; FvIAA14 : 1534915 ; FvIAA15 : 1535927 ; FvIAA16 : 1540186 ; FvIAA17 : 1540651 ; FvIAA18 : 1541688 ; FvIAA19 : 1541690 ; FvIAA20 : 1517318 ; FvIAA21 : 1536988 ; FvIAA22 : 1518131 ; FvIAA23 : 1514680 ; FvIAA24 : 1531884 ; FvIAA25 : 1515115 ; FvIAA26 : 1540036 ; FvIAA32 : 1512801

*Solanum lycopersisum*

SlIAA1: SGN-P670503; SlIAA3: SGN-P669106; SlIAA4 : SGN-P670838; SlIAA6 : SGN-P668800; SlIAA7 : SGN-P670266; SlIAA8 : SGN-P670660; SlIAA9 : SGN-P660129; SlIAA12: SGN-P670882; SlIAA13: SGN-P670451; SlIAA14: SGN-P670709; SlIAA16: SGN-P671234; SlIAA17: SGN-P683983; SlIAA26: SGN-P664563; SlIAA27: SGN-P672765; SlIAA29: SGN-P660244
