# Supplementary material for: A genomics approach to understanding the role of auxin in apple (Malus x domestica) fruit size control
Source: BMC Plant Biol. 2012 Jan 13;12:7. doi: 10.1186/1471-2229-12-7 (PMC3398290; doi:10.1186/1471-2229-12-7)
Supplement: Additional file 3 — Phylogenetic trees for PIN, GH3 and Aux/IAA class of genes. Protein sequences of PIN, GH3, Aux/IAA from apple (green), strawberry (lilac), Arabidopsis (black) and tomato (red) were aligned using MUSCLE and phylogenetic trees were built using neighbour joining. Bootstraps of 1000 iterations are given. At: Arabidopsis thaliana, Fv: Fragaria vesca, Md: Malus x domestica, Pp: Physcomitrella patens, Sl: Solanum lycopersicu. [file 1471-2229-12-7-S3.DOCX]

**Phylogenetic trees for PIN, GH3 (groupII) and Aux/IAA gene families**

Protein alignment and phylogenetic trees were obtained as described in material and methods

**PIN phylogenetic tree**


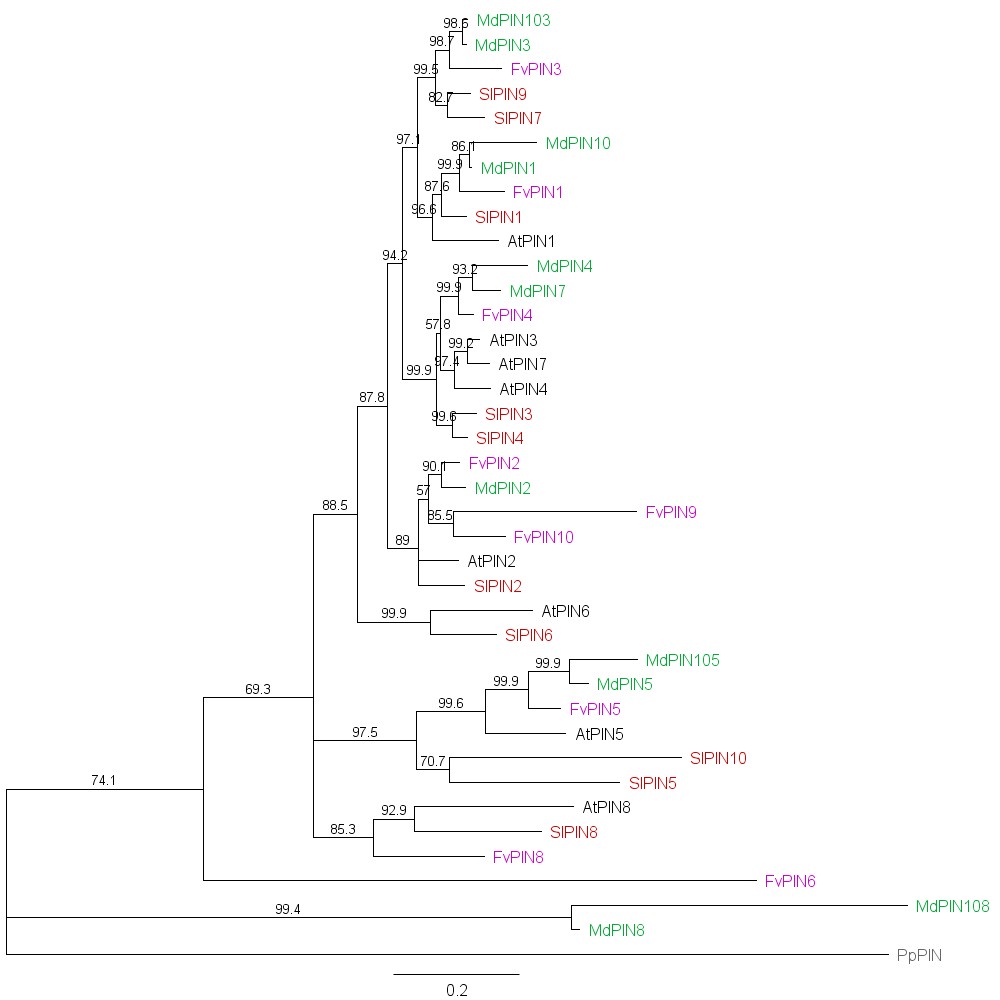


**GH3 (group II) phylogenetic tree**


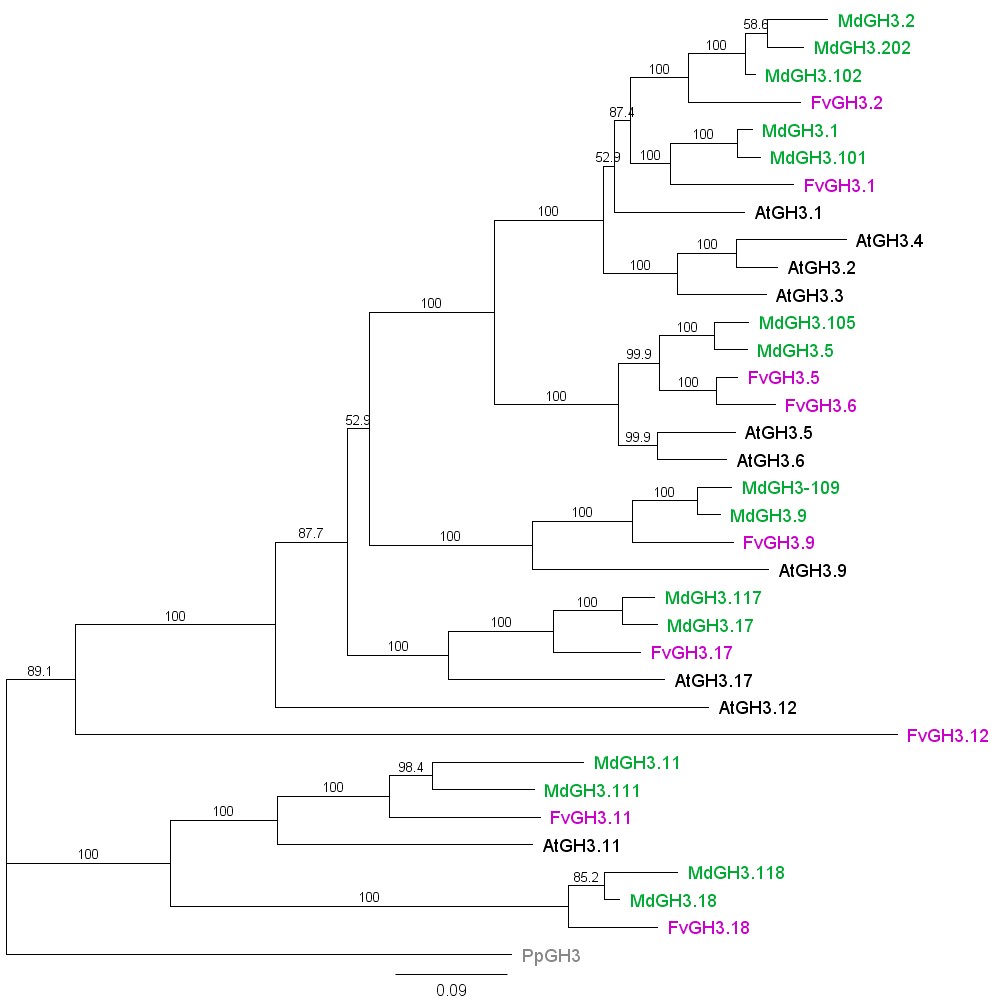


**Aux/IAA phylogenetic tree**

**
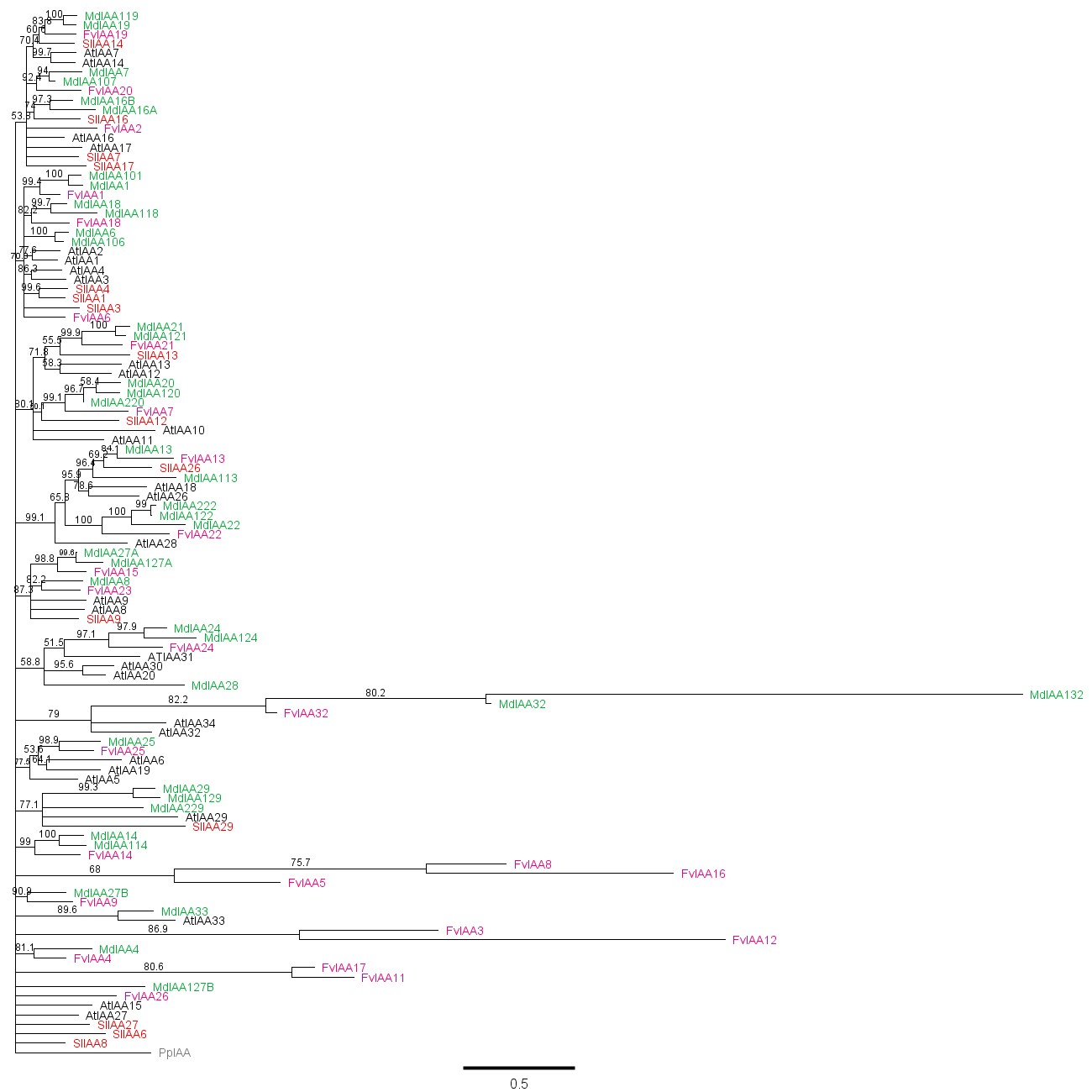
**
