# Supplementary material for: A genomics approach to understanding the role of auxin in apple (Malus x domestica) fruit size control
Source: BMC Plant Biol. 2012 Jan 13;12:7. doi: 10.1186/1471-2229-12-7 (PMC3398290; doi:10.1186/1471-2229-12-7)
Supplement: Additional file 5 — Mapping Quantitative Trait Loci (QTL)s for fruit weight in RGxBB and STKxGS. [file 1471-2229-12-7-S5.PPT]

## Slide 1
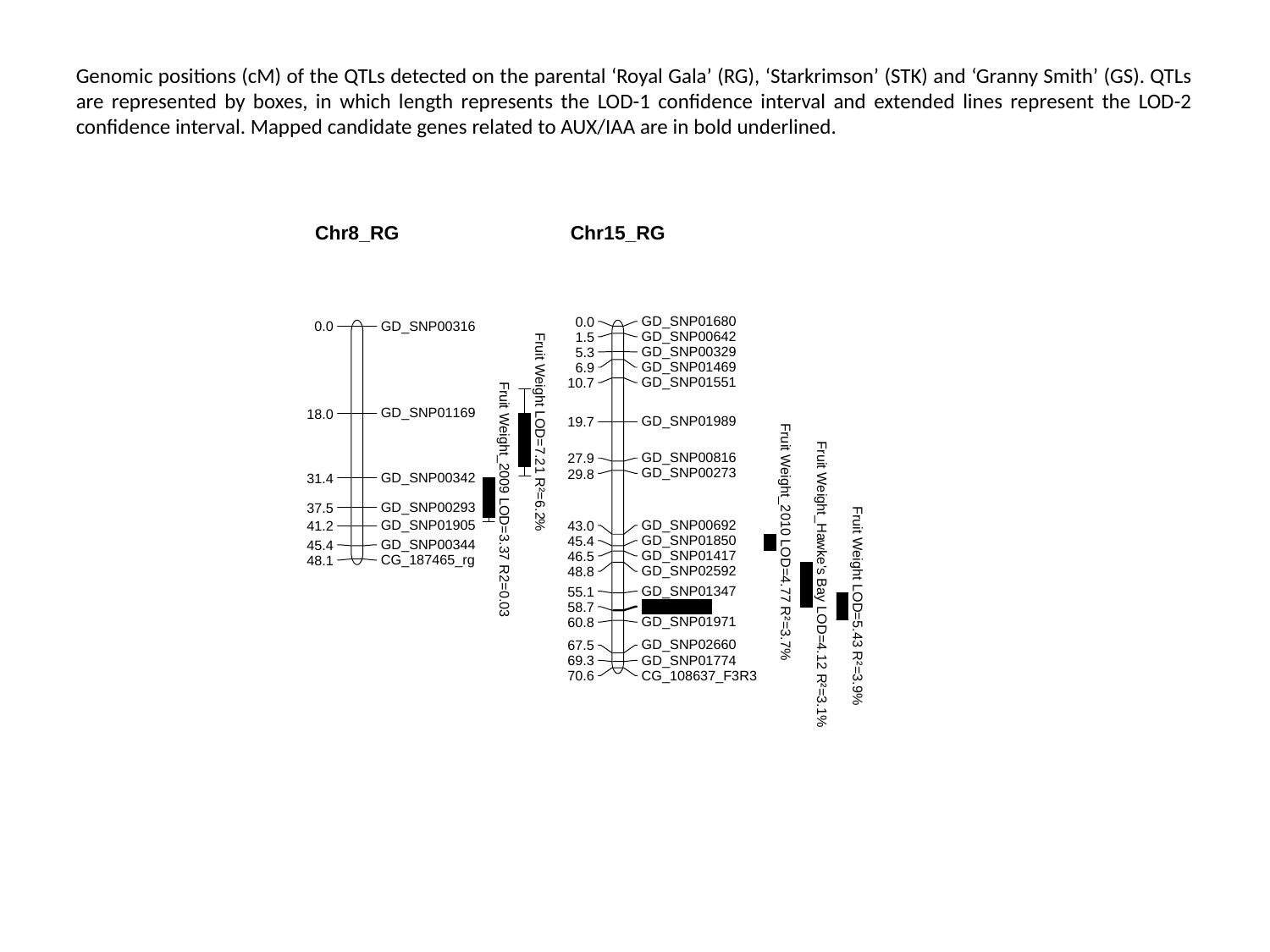

# Genomic positions (cM) of the QTLs detected on the parental ‘Royal Gala’ (RG), ‘Starkrimson’ (STK) and ‘Granny Smith’ (GS). QTLs are represented by boxes, in which length represents the LOD-1 confidence interval and extended lines represent the LOD-2 confidence interval. Mapped candidate genes related to AUX/IAA are in bold underlined.

## Slide 2
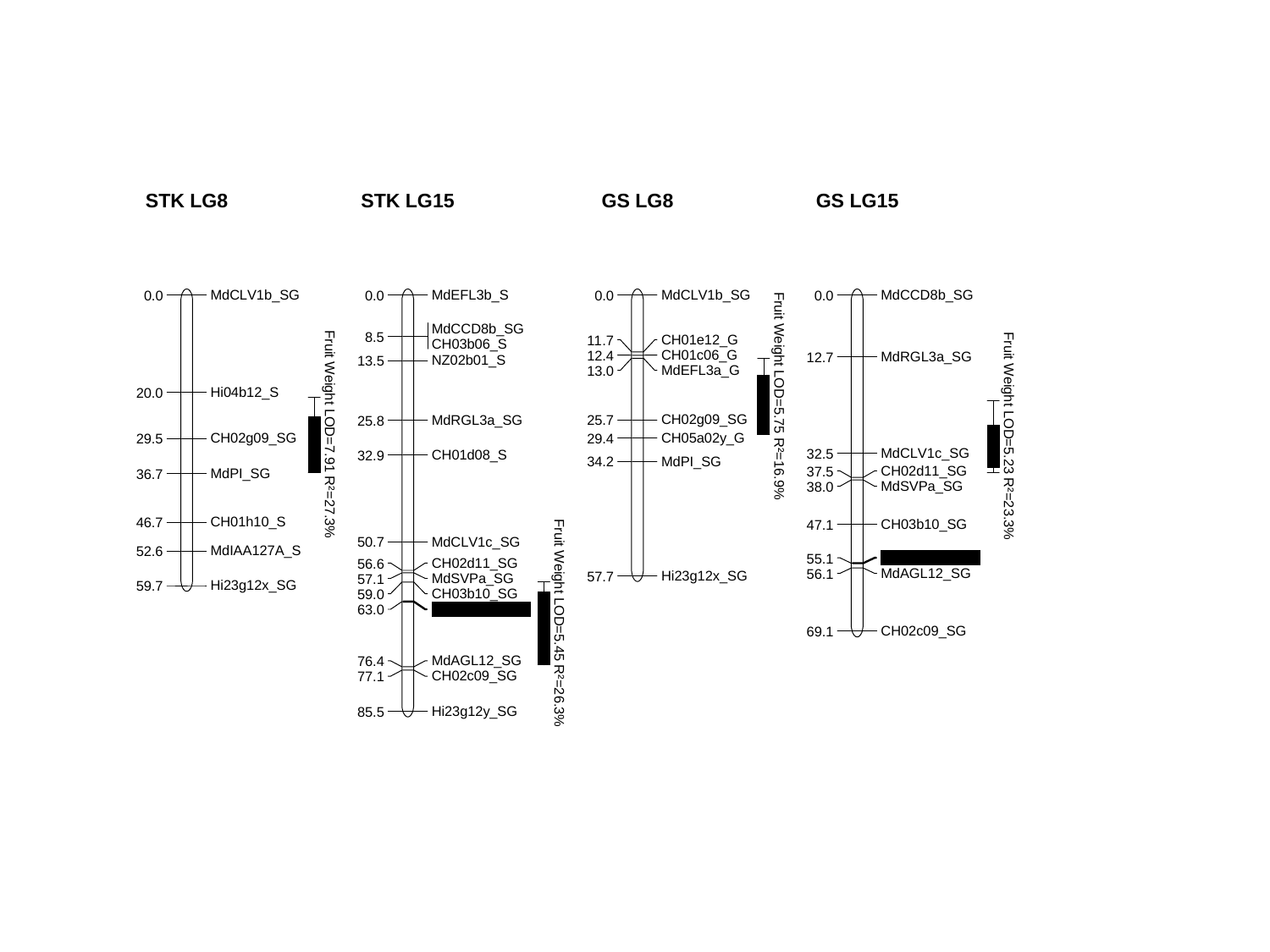

## Slide 3
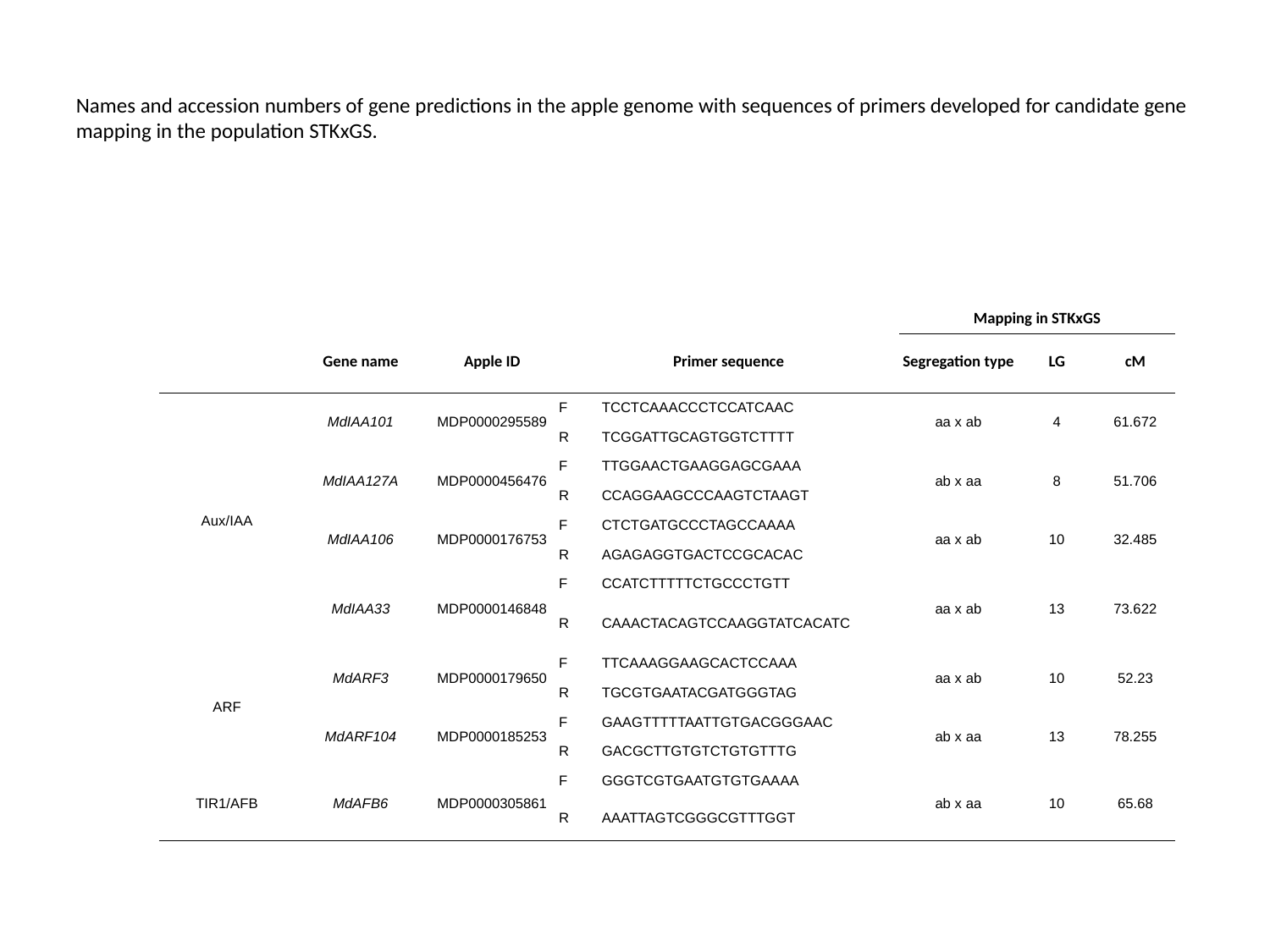

# Names and accession numbers of gene predictions in the apple genome with sequences of primers developed for candidate gene mapping in the population STKxGS.
| | | | | | Mapping in STKxGS | | |
| --- | --- | --- | --- | --- | --- | --- | --- |
| | Gene name | Apple ID | Primer sequence | | Segregation type | LG | cM |
| Aux/IAA | MdIAA101 | MDP0000295589 | F | TCCTCAAACCCTCCATCAAC | aa x ab | 4 | 61.672 |
| | | | R | TCGGATTGCAGTGGTCTTTT | | | |
| | MdIAA127A | MDP0000456476 | F | TTGGAACTGAAGGAGCGAAA | ab x aa | 8 | 51.706 |
| | | | R | CCAGGAAGCCCAAGTCTAAGT | | | |
| | MdIAA106 | MDP0000176753 | F | CTCTGATGCCCTAGCCAAAA | aa x ab | 10 | 32.485 |
| | | | R | AGAGAGGTGACTCCGCACAC | | | |
| | MdIAA33 | MDP0000146848 | F | CCATCTTTTTCTGCCCTGTT | aa x ab | 13 | 73.622 |
| | | | R | CAAACTACAGTCCAAGGTATCACATC | | | |
| ARF | MdARF3 | MDP0000179650 | F | TTCAAAGGAAGCACTCCAAA | aa x ab | 10 | 52.23 |
| | | | R | TGCGTGAATACGATGGGTAG | | | |
| | MdARF104 | MDP0000185253 | F | GAAGTTTTTAATTGTGACGGGAAC | ab x aa | 13 | 78.255 |
| | | | R | GACGCTTGTGTCTGTGTTTG | | | |
| TIR1/AFB | MdAFB6 | MDP0000305861 | F | GGGTCGTGAATGTGTGAAAA | ab x aa | 10 | 65.68 |
| | | | R | AAATTAGTCGGGCGTTTGGT | | | |
